# Lipid Related Genes Altered in NASH Connect Inflammation in Liver Pathogenesis Progression to HCC: A Canonical Pathway

**DOI:** 10.3390/ijms20225594

**Published:** 2019-11-08

**Authors:** Christophe Desterke, Franck Chiappini

**Affiliations:** 1 Inserm, UMR-S 935, Université Paris Sud, F-94800 Villejuif, France; christophe.desterke@inserm.fr; 2 Laboratoire Croissance, Régénération, Réparation et Régénération Tissulaires (CRRET)/ EAC CNRS 7149, Univ Paris-Est Créteil (UPEC), F-94010 Créteil, France

**Keywords:** NAFLD, NASH, canonical pathway, text-mining, lipids, inflammation

## Abstract

Nonalcoholic steatohepatitis (NASH) is becoming a public health problem worldwide. While the number of research studies on NASH progression rises every year, sometime their findings are controversial. To identify the most important and commonly described findings related to NASH progression, we used an original bioinformatics, integrative, text-mining approach that combines PubMed database querying and available gene expression omnibus dataset. We have identified a signature of 25 genes that are commonly found to be dysregulated during steatosis progression to NASH and cancer. These genes are implicated in lipid metabolism, insulin resistance, inflammation, and cancer. They are functionally connected, forming the basis necessary for steatosis progression to NASH and further progression to hepatocellular carcinoma (HCC). We also show that five of the identified genes have genome alterations present in HCC patients. The patients with these genes associated to genome alteration are associated with a poor prognosis. In conclusion, using an integrative literature- and data-mining approach, we have identified and described a canonical pathway underlying progression of NASH. Other parameters (e.g., polymorphisms) can be added to this pathway that also contribute to the progression of the disease to cancer. This work improved our understanding of the molecular basis of NASH progression and will help to develop new therapeutic approaches.

## 1. Introduction

Liver is a major integrator of metabolism and plays a key role in lipid metabolism including fatty acid oxidation, lipogenesis, cholesterol synthesis, and production of triglycerides and lipoproteins [1,2,3]. A variety of conditions result in dysregulation of lipid metabolism which leads to fat accumulation in the liver and then to nonalcoholic fatty liver disease (NAFLD). NAFLD is a pathological condition, exhibiting a wide range of lesions starting with the accumulation of lipid droplets in the liver also known as hepatic steatosis or nonalcoholic fatty liver (NAFL). NAFL may progress to nonalcoholic steatohepatitis (NASH) and then to hepatocellular carcinoma (HCC) [4,5,6]. Furthermore, NAFLD is a systemic disease associated with obesity, type 2 diabetes mellitus, and metabolic syndrome [7,8,9,10] that are dramatically increasing worldwide and currently present a major public health problem [11,12,13,14].

The hallmark of NAFLD is the intra-cellular accumulation of lipids, particularly triglycerides cholesteryl esters and phospholipids resulting in the formation of lipid droplets in hepatocytes [15,16,17]. Fatty liver is a reversible and asymptomatic lesion that has long been considered benign. However, we have previously demonstrated that progressive intrahepatic inflammation could be present from the beginning of the disease, potentially driven by the specific types of accumulated lipids [18]. Certain lipids have been shown to be specifically linked to the inflammatory process and NASH progression [19,20,21,22,23]. Therefore, the progression of fatty liver to NASH is related to the lipid composition [19,24,25,26,27,28]. Our previous studies revealed alterations in homeostasis of triglycerides, cholesterol, phospholipids, and long-chain fatty acids during the progression of NASH [19,20]. Indeed, an increase in lipid species such as saturated fatty acids and phospholipids, as well as disturbances in ceramide-signaling or alterations in cholesterol content are associated with pro-inflammatory and pro-apoptotic properties [16,19,20,29,30,31,32,33,34]. Moreover, alterations in lipid metabolism during NASH progression have been associated with gene expression changes and single nucleotide polymorphisms in genes involved not only in lipid metabolism but also in genes associated with inflammatory and cancerous processes [18,19,28,35,36,37,38,39,40,41,42,43,44,45,46].

Since 1980, when Ludwig and colleagues at Mayo Clinic (Rochester, MN) described NASH for the first time [47], NASH and more generally NAFLD have received increasing attention over the years. More than 2000 manuscripts on “NASH” and “lipid” have been published in PubMed and the number of studies is growing. However, the pathogenesis of NAFLD remains incompletely understood, especially the conditions that lead to progression from NAFL to NASH, and then to cirrhosis or cancer, but only in a subset of patients.

Scientific literature has become the key distribution channel for novel findings and hypotheses from the exponential number of research studies in this area. As a result of the continuous increase in the number of publications, retrieving relevant scientific information and identifying connections between pieces of scientific knowledge have become challenging but necessary tasks. As a consequence, automated literature analysis is now frequently a part of complex biomedical research and often delivers crucial background knowledge [48]. A plethora of publicly available biomedical resources do currently exist and are constantly increasing. In parallel, specialized repositories have been developed, indexing numerous clinical and biomedical tools. Natural Language Processing research in the clinical domain has been active since the 1960s. In addition to maintaining the GenBank® nucleic acid sequence database, the National Center for Biotechnology Information (NCBI) provides analysis and retrieval resources for the data in GenBank® and other biological data made available through the NCBI website [49,50,51,52,53,54].

Here we used text-mining and data-mining bioinformatic approaches by investigating Medical Subject Headings (MeSH) and gene expression omnibus dataset (NCBI) combined with different databases (e.g., Kyoto Encyclopedia Gene and Genome/KEGG or InnateDB) to determine specific and global mechanisms involved in NASH progression. We identified a set of at least twenty-five (*n* = 25) genes that play a role either in lipid synthesis and excretion, inflammatory cells recruitment and activation, insulin signaling pathway, or hepatic cancer development. These genes are orchestrated by a new player *YWHAZ* and they are dysregulated in most cases of pathological NAFL progression. Importantly, for the first time these combined approaches used together connected mechanisms that can be described as the core pathway, the “canonical pathway”, involved in progression of NASH from hepatic steatosis and insulin resistance to HCC.

## 2. Results

### 2.1. A Subset of Lipid-Related Genes is Differentially Expressed in NASH vs Healthy Obese Human Liver Samples

**Figure 1:**
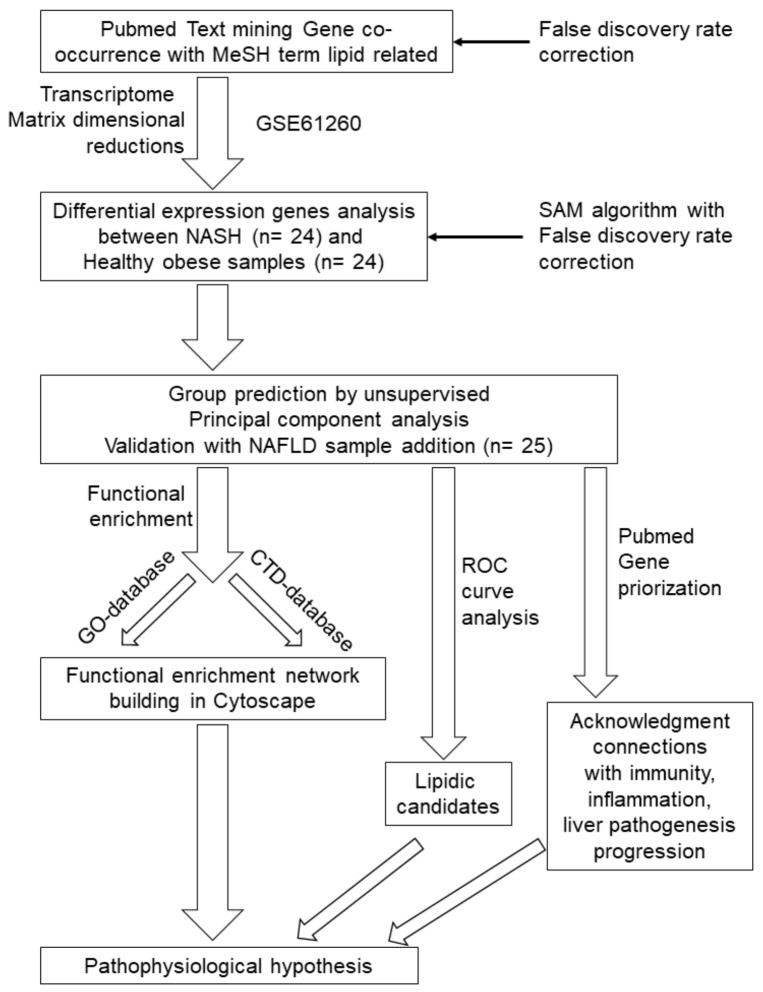
Workflow of analysis implemented from text-mining integration in transcriptome to pathophysiological hypothesis. CTD: comparative Toxicogenomics database; GEO: gene expression omnibus; GSE: genomic spatial event database, MeSH: medical subject headings; NAFLD: nonalcoholic fatty liver disease; NASH nonalcoholic steatohepatitis; ROC: receiver operating characteristic; SAM: statistical analysis of microarray.

**Figure 2:**
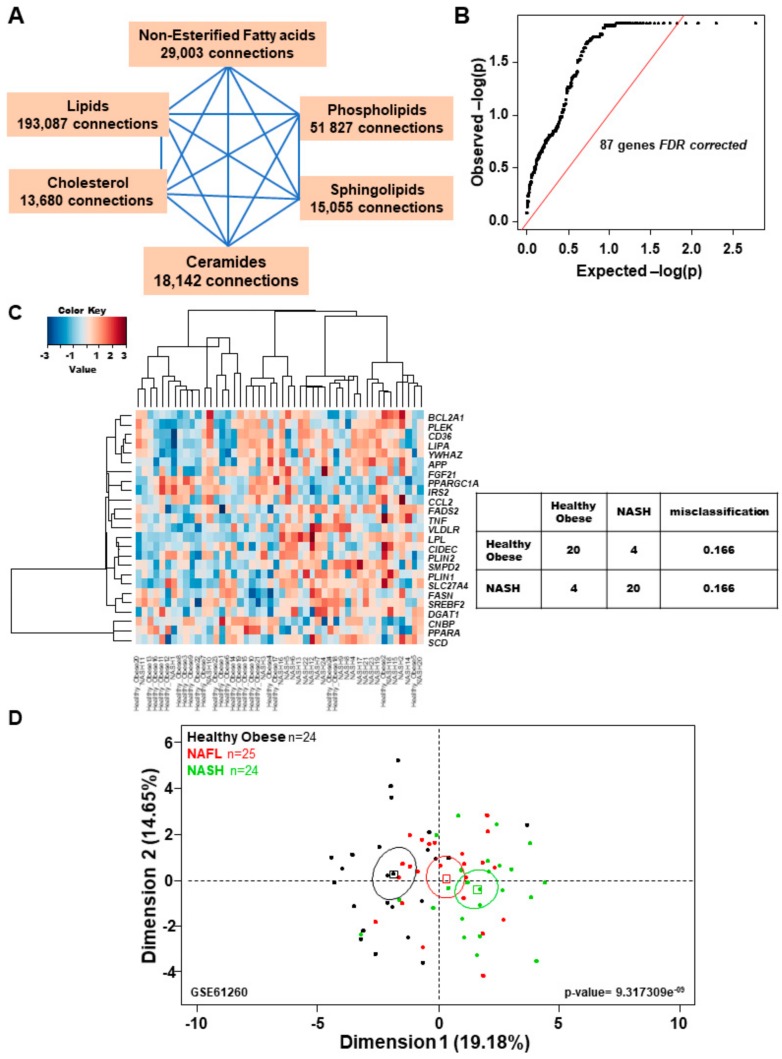
Lipid-related genes differentially expressed between NASH and healthy obese liver samples. (**A**) Text-mining summary of connections observed between genes and lipid terms, number of gene co-occurrence of gene symbol with language terms in scientific literature. (**B**) qqplot of *q*-values obtained by false discovery rate correction of text-mining results (87 genes are still significant after correction, *q*-values < 0.05). (**C**) Expression heatmap of lipid related genes found differential expressed between NASH liver samples (*n* = 24) and Healthy obese liver samples (*n* = 24) in transcriptome dataset GSE21260 (**D**) unsupervised principal component analysis performed with lipid related genes found differentially expressed between NASH and Healthy obese in GSE21260 and impact of their prediction to predicted NAFL samples (*n* = 23), p-value was calculated by correlation of sample group discrimination on first principal axis.

Natural language processing is performed to discover semantic relationships with scientific literature [51], and to connect literature to databases like RefSeq gene symbol identifiers [55]. The most important resource for text-mining applications is currently the PubMed database developed by the National Center for Biotechnology Information (NCBI) at the National Library of Medicine (NLM) (https://www.nlm.nih.gov/pubmed) [49,50,52,53,54,56]. Text-mining analysis by connecting the PubMed database using MeSH described by the workflow on Figure 1 allowed us to find 320,794 co-occurrence connections between gene symbol and lipid-related terms: “Non-esterified Fatty acids” (29,003connections), “lipids” (193,087 connections), “cholesterol” (13,680 connections), “ceramides” (18,142 connections), “sphingolipids”(51,827 connections), and “phospholipids” (15,055 connections) related to “NASH” (Figure 2A).

False discovery rate correction performed on genes selected by text-mining allowed us to find 87 lipid-related genes that show significant association in scientific literature (Figure 2B and Supplementary Datasets Table S1 online). Mathematical dimensional reduction of matrix in transcriptome analysis, such as using gene set enrichment analysis (GSEA), allowed us to improve discovery by reducing the false positive discovery rate [57]. We applied text-mining dimensional reduction on normalized gene expression matrix from dataset GSE61260 in order to find differentially expressed lipid-related genes with improved accuracy. Significance Analysis for Microarray (SAM) algorithm was employed on reduced matrix (87 genes × 48 samples) between liver samples from NASH patients (*n* = 24) and liver samples from healthy obese (HO) subjects. Twenty-five genes were found to be differentially expressed between samples with an FDR threshold set to less than five percent (FDR < 5%; Supplementary Datasets Table S2 online). Expression heatmap revealed that 22 of 25 differentially expressed lipid-related genes were over-expressed in NASH liver samples compared to healthy obese liver samples and three were down-regulated (*PPARA*, *PPARGC1A*, and *CNPB*) associated with misclassification error rate of 16.6% (Figure 2C). *LPL* gene encoding for lipoprotein lipase was the most over-expressed gene in NASH with a fold change of +1.93, followed by chemokine *CCL2* (fold change = +1.61) and the enzyme *FADS2* (fatty acid desaturase 2, fold change = +1.57) as summarized in Supplementary Datasets Table S2 online.

Transcriptome expression matrix was increased by addition of 25 liver samples of patients with NAFL (resulting lipid related matrix dimensions: 87 genes × 73 samples). Principal component analysis (PCA) was performed on this increased expression matrix. Text-mining for lipid related genes allowed us to significantly and progressively discriminate between liver samples from HO subjects, NAFL and NASH patients (*p*-value = 9.32 × 10^-9^, Figure 2D) on the first principal axis (i.e., first dimension) of the unsupervised analysis.

Thus, with this first approach we identified 25 genes that can be used to discriminate between three groups of patients: HO subjects, obese with NAFL or NASH patients. Moreover, this analysis identified a precancerous pathway led by *YWHAZ*.

### 2.2. NASH is Associated with Impaired Function of Genes Implicated in Lipid Metabolism, Insulin-Resistance, Inflammation and Tumorigenesis

**Figure 3:**
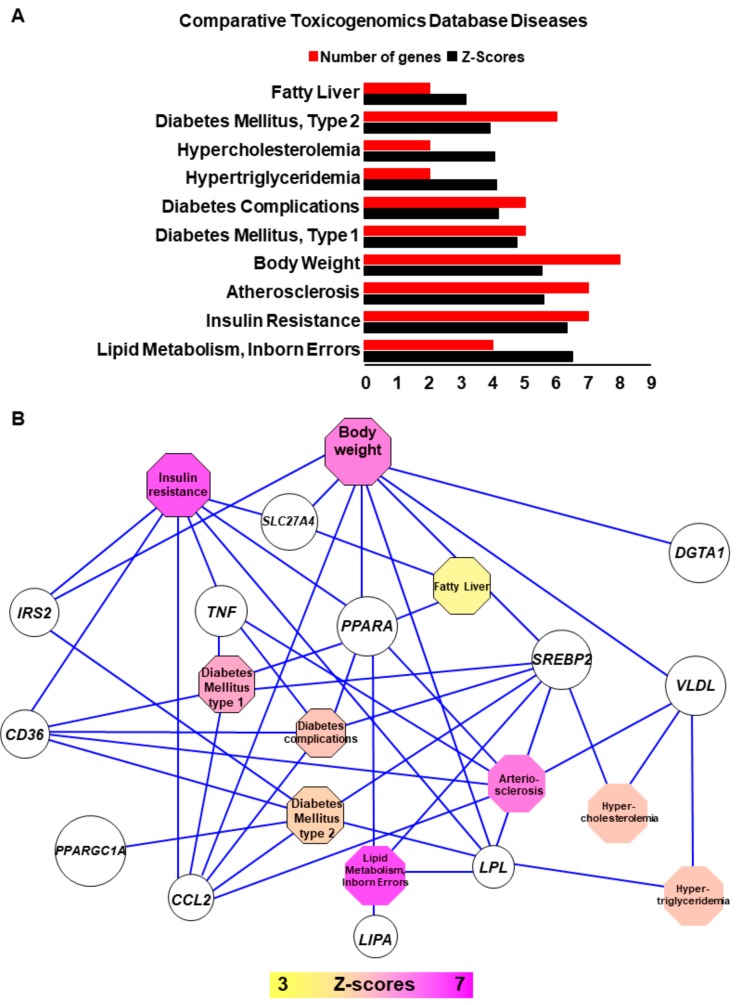
Disease enrichment network of lipid related genes deregulated in NASH liver samples. (**A**) Bar plot of functional enrichment performed with lipid related genes deregulated in NASH on CTD disease database: red bars represent number of genes implicated by function and blue bars respective Z-scores of the enrichments. (**B**) Functional enrichment network performed with lipid related genes deregulated in NASH (CTD disease database).

We further examined the relationship between lipid genes and HCC, and found that differentially expressed lipid-related genes (Supplementary Datasets Table S2 online) were enriched on the Comparative Toxicogenomics Database which connects genes to disease phenotypes [58]. As expected, this enrichment confirmed that these genes have a well association with phenotypic manifestations of NASH such as lipid metabolism perturbations linked to fatty liver, hypercholesterolemia, and hypertriglyceridemia. They are also associated with Type 1 and Type 2 diabetes, insulin resistance, and impairment in clinical parameters such as body weight and atherosclerosis (Figure 3A). These results suggest that the lipid text-mining approach for analysis of the transcriptome that we developed is well adapted to study the altered gene-expression signature of NASH (Figure 3B).

**Figure 4:**
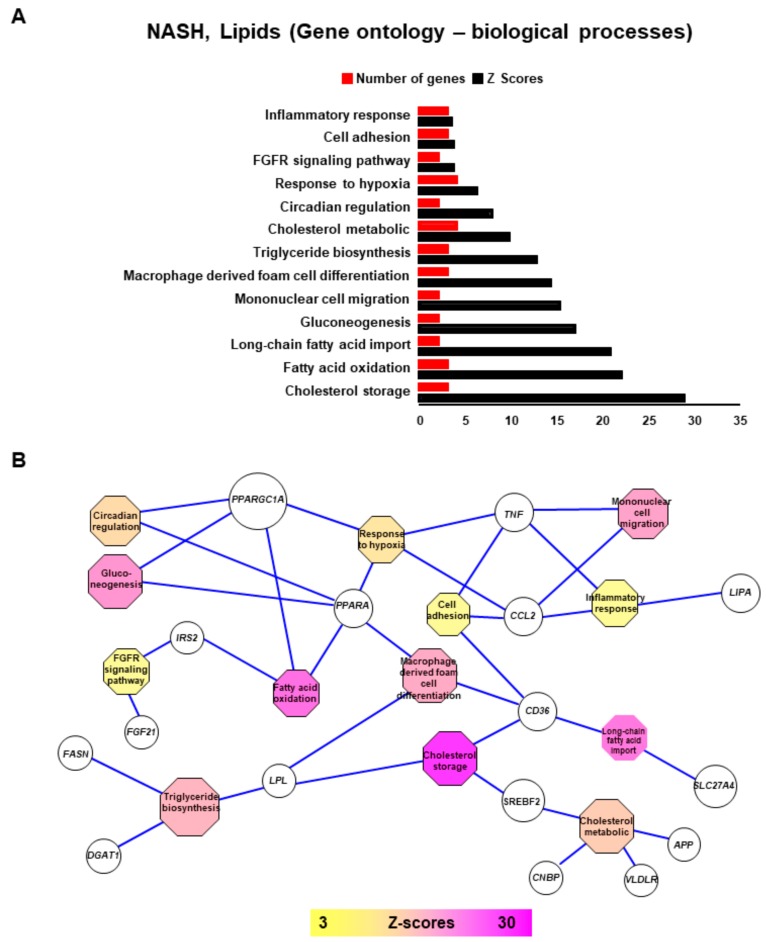
Ontologic functional enrichment network of lipid-related genes deregulated in NASH liver samples. (**A**) Bar plot of functional enrichment performed with lipid related genes deregulated in NASH on Gene Ontology Biological Process database: red bars represent number of genes implicated by function and blue bars respective Z-scores of the enrichments. (**B**) Functional enrichment network performed with lipid related genes deregulated in NASH (Gene ontology biological process).

The 25 differentially expressed lipid-related genes identified earlier (Supplementary Datasets Table S2 online) were also analyzed by their functionalities using Gene Ontology Biological Process database. As expected, functional enrichment in this database revealed that these genes are mostly implicated in lipid metabolism, especially cholesterol storage (*LPL*, *CD36*, and transcription factor *SREBF2* were significantly up-regulated), fatty acid oxidation (*PPARA* and *PPARGC1A* were down-regulated), long-chain fatty acid import (transporters *SLC27A4* and *CD36* were up-regulated), and triglyceride biosynthesis (*FASN*, *DGAT1*, and *LPL* were all upregulated) as summarized in Figure 4.

Interestingly, we observed that some lipid-related genes altered in NASH are also enriched in other important biological pathways such as circadian rhythm (Figure 4A) which is represented by down-regulation of PPAR pathway (*PPARA* and *PPARGC1A*). Down regulation of *PPARA* and *PPARGC1A* genes also share other functionalities such as response to hypoxia, gluconeogenesis, and mitochondrial functions such as regulation of β-oxidation. These analyses also highlighted that lipid related genes altered in NASH samples affect processes linked to monocyte/macrophage infiltration into the tissue and to inflammation (Figure 4B), especially by up-regulating cytokines/chemokines such as *TNF* and *CCL2*. At the same time *CD36*, a receptor of oxLDL, led to increased flux of phospholipids and long-chain fatty acids into the hepatocytes increasing formation of lipid droplets and ceramides that are chemo-attractants for macrophages (Figure 3 and Figure 4).

Thus, this analysis showed the hard connection between early non-specific inflammation processes and the progression of NAFL to NASH.

### 2.3. Connecting Lipid Related Genes Altered in NASH to Immunity, Inflammation and Liver Pathogenesis Progression

**Figure 5:**
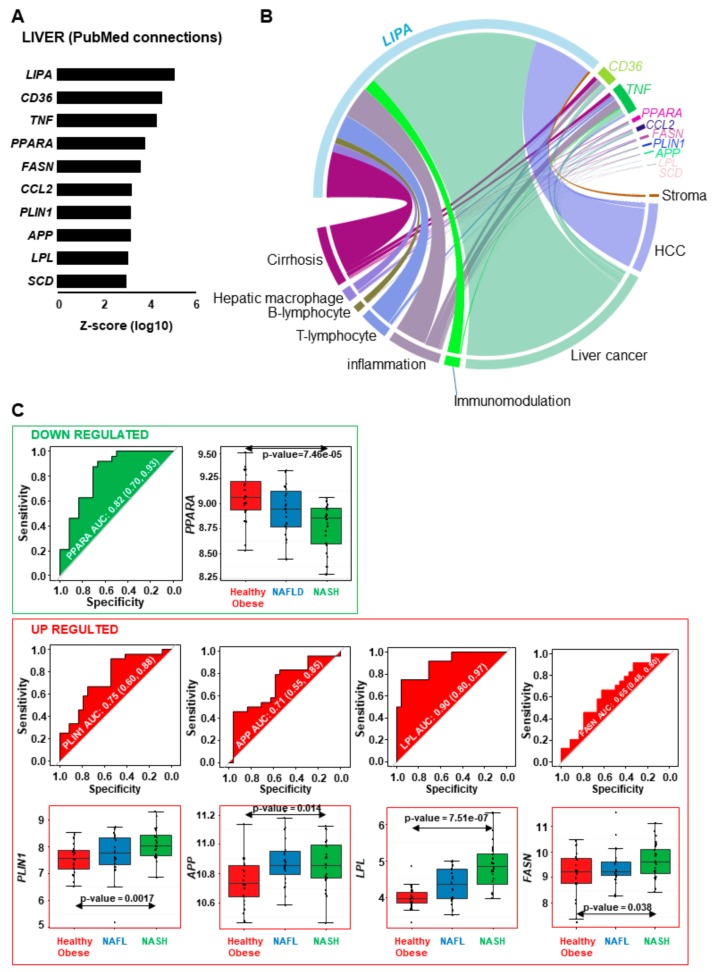
Lipid related genes found to be deregulated in NASH liver samples are also implicated in inflammation, immunity and liver pathogenesis progression. (**A**) Bar plot of connection numbers obtained by text-mining on first prioritization term: liver. (**B**) Circoplot representing connection numbers during text-mining prioritization between lipid related genes altered in NASH and terms around immunity, inflammation and liver disease progression. (**C**) individual ROC-curves (AUC and its confident intervals) testing expression regulation between NASH and healthy obese liver samples for lipid related genes and prioritized on inflammation-immunity and liver pathogenesis progression, Expression boxplot with inclusion of NAFL samples (*p*-value was calculated by two-tailed Student *t*-test between NASH and Healthy obese liver samples).

As observed above in functional enrichment performed with Gene Ontology Biological Process, lipid-related genes altered in NASH also showed implication of monocyte/macrophage cells and inflammation (Figure 4). Responses to hypoxia, gluconeogenesis, and circadian rhythm were also found to be affected by down-regulation of *PPAR* genes. So, these results showed that inflammation and progression of liver pathogenesis could be affected by lipid related genes in NASH, as demonstrated above. In order to cross-reference this information, we wanted to verify the role these molecules may play in immunity, inflammation, and liver pathogenesis progression through the PubMed database. Candidate gene prioritization approach allowed to focus on important affected genes with literature relevance [59]. Connection with NCBI database allowed to select the 10 best lipid-related genes that are important in the liver (Figure 5A and Supplementary Datasets Table S3 online).

For these 10 liver-related genes, we applied PubMed filtering with secondary terms related to immunity, inflammation, and liver pathogenesis progression such as stroma, hepatocellular carcinoma, liver cancer, cirrhosis, hepatic macrophage, B-lymphocyte, T-lymphocyte, inflammation, and immunomodulation. This analysis showed a good literature prioritization for *CD36* and *TNF*, and also highlighted *LIPA* gene which is well-known to play a role in liver pathophysiology, (Figure 5B and Supplementary Datasets Table S4 online). Among these 10 prioritized genes, individual ROC-curves and expression boxplots (Figure 5C) were performed with transcriptome matrix for genes that were found also deregulated in NASH liver samples (Supplementary Datasets Table S2 online). One of them, *PPARA* was found to be down-regulated in NASH samples as compared to healthy obese samples. *PPARA* had an area under curve (AUC) of 0.82 (Figure 5C) and a significant individual down-regulation in NASH compared to HO subjects (two-tailed Student *t*-test *p*-value = 7.46 × 10^-5^). Among up-regulated prioritized genes, we found *PLIN1* with an AUC of 0.75 (two-tailed Student *t*-test *p*-value = 0.00017), *APP* with an AUC of 0.71 (two-tailed Student *t*-test *p*-value = 0.014), *LPL* with an AUC of 0.90 (two-tailed Student *t*-test *p*-value = 7.51 × 10^-7^), and *FASN* with an AUC of 0.65 (two-tailed Student *t*-test *p*-value = 0.038).

Taken together, depending on the relative contribution of *LIPA* and the other nine genes’ expression levels, the fate of the disease will be inflammatory response associated to *CD36*, *LPL*, and *SCD* leading to NASH progression into liver cancer or cirrhosis and HCC as shown in Figure 5B.

### 2.4. The 14-3-3 Protein Family is the Cornerstone between Dysregulated Lipid Metabolism, Inflammatory and Insulin Pathways during NASH Progression to HCC

Thus, with the first analysis, genes implicated in inflammation, lipid metabolism, and progression to cancer were highlighted, and the second analysis showed connection between lipid dysregulation and insulin resistance and inflammation. We then focused on the *YWHAZ* gene identified in Figure 2A, a member of 14-3-3 protein family, which has been associated with liver cancer [60].

**Figure 6:**
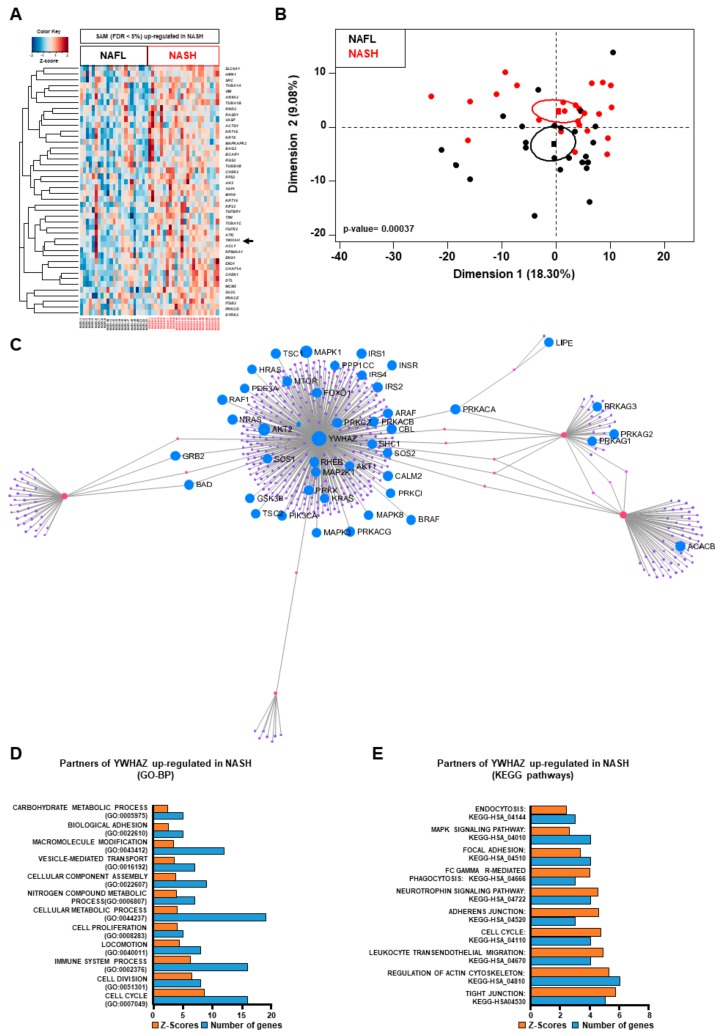
14-3-3 protein family is at the center of the metabolic, inflammatory and dysregulated insulin pathways. (**A**) Significance Analysis of Microarray identified 44 genes up-regulated linked with YWHAH gene belonging to 14-3-3 protein family and represented by heatmap discriminating NAFL and NASH patients with FDR < 5%. (**B**) Principal component analysis discriminates NAFLD and NASH patients based on 44 genes up-regulated with a global *p*-value of 0.00037. (**C**) YWHAZ a 14-3-3 protein family and its protein partners (*n* = 399) linked to insulin signaling (44 proteins) based on Kyoto Encyclopedia Gene and Genome (KEGG) associated to FDR of *q*-value = 2 × 10^-19^. Partners of YWHAZ up-regulated in NASH based on (**D**) GO-BP and (**E**) KEGG pathways. FDR: false discovery rate.

A total of 399 YWHAZ-protein partners were identified based on InnateDB database. Significance analysis of microarray (SAM) of the 399 protein partners performed on GSE61260 [61] identified 44 genes that discriminated between NAFL patients and NASH patients as shown by the heatmap (Figure 6A and Supplementary Datasets Table S5 online) and by PCA analysis (Figure 6B).

The 44 genes are up-regulated in the group of NASH patients demonstrating their connection (Figure 6C) with inflammatory processes, cell proliferation, metabolism, and especially cell–cell adhesion based on GO-BP and KEGG pathway analysis (Figure 6D and 6E). One of these 44 genes, *YWHAH* (Figure 6A and Supplementary Datasets Table S5 online) belongs also to the 14-3-3 proteins family and it has also been associated to insulin function [62] and liver cancer [63].

Taken together, these data show that 14-3-3 protein family has an important role in the progression of NAFL to NASH through the dysregulation of metabolism and inflammatory processes associated with cell proliferation and dysregulation in cell–cell adhesion, making the link to the progression to HCC.

### 2.5. Connecting Lipid Dysregulation, Insulin Resistance, Inflammatory Processes and HCC: a NASH Canonical Pathway

**Figure 7:**
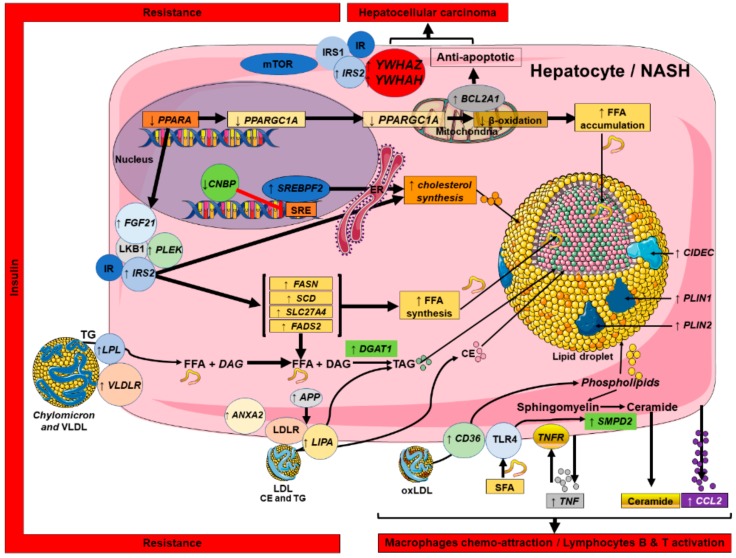
NASH canonical pathway defined based on the 25 genes that were found using text-mining analysis. Draw of pathophysiologic hypothesis connected to altered-lipid related genes expression in liver samples of NASH and associated to immunity, inflammation and liver pathogenesis progression.

The main results of these analyses are summarized in the Figure 7 and connect lipid dysregulation, insulin resistance, inflammatory processes, and HCC development based on the 25 genes found after text-mining and in silico analyses.

A decrease in *PPARA* leads to an increase in *FGF21* [64], *PLEK*, and *IRS2* which will result in the increase of *FASN*, *SCD*, *SLC27A2*, and *FADS2* all of which participate in de novo fatty acid synthesis.

At the same time, expression of *CNPB*, an inhibitor of *SREBP2* expression, decreases. This leads to an increase in *SREBP2* expression and results in increased cholesterol synthesis.

Also, *LPL* and *VLDR* expression are increased (likely due to the lipid-rich diet), leading to an accumulation of lipids in the cell. In addition, expression of *APP* and *LIPA* is increased, promoting triglyceride synthesis and release of cholesteryl ester. Altogether, this leads to an increase in expression of genes implicated in the synthesis of droplets such as *CIDEC*, *PLIN1*, and *PLIN2* to create lipid droplets. In parallel, we showed increase in expression of *CD36*, *SMPD2*, and *CCL2* which control phospholipid metabolism and vesicle assembly and are also implicated in inflammatory processes and inflammatory cell chemo-attraction. Finally, the down-regulation of *PPARA* leads to a decrease in *PPARG1A* that leads to a decrease in *PPARGC1A* (i.e., *PGC1*-α), a major player in the β-oxidation, thus contributing to an increased accumulation of FFA in the cell by disturbing mitochondrial function.

Our analysis also showed an increase in *BCL2A1*, an anti-apoptotic player, usually implicated in cancer progression. In association with an increase in expression of *YWHAZ* and *YWHAH*, *IRS2* is also up-regulated. This group of genes participates in development of the insulin resistance as well as hepatocellular carcinoma.

For the first time, using text-mining, bioinformatics, and statistics, a precise picture of the progression of NAFL to NASH and then HCC has been shown, describing a canonical pathway shown in Figure 7.

### 2.6. Confirmation of the NASH Canonical Pathway Using Independent Illumina Gene Expression Beadchip 

To confirm our findings and demonstrate that the “canonical signature” based on the 25 genes identified after the text-mining approach is applicable to other groups of NAFL and NASH patients, we used these set of genes to a new group of healthy control subjects, NAFL, and NASH patients, for which gene expression analyses were performed using a completely different approach than that used to establish this signature. The gene expression analyses were performed in 44 human liver surgical samples (normal *n* = 13; steatosis *n* = 19; steatohepatitis *n* = 12), which were processed with Illumina HumanWG-6 v3.0 expression beadchip technology and referenced as GSE33814 [65]. Two genes, *PLIN1* and *PLIN2* implicated in lipid droplet synthesis, were not annotated in the Illumina beadchip.

**Figure 8:**
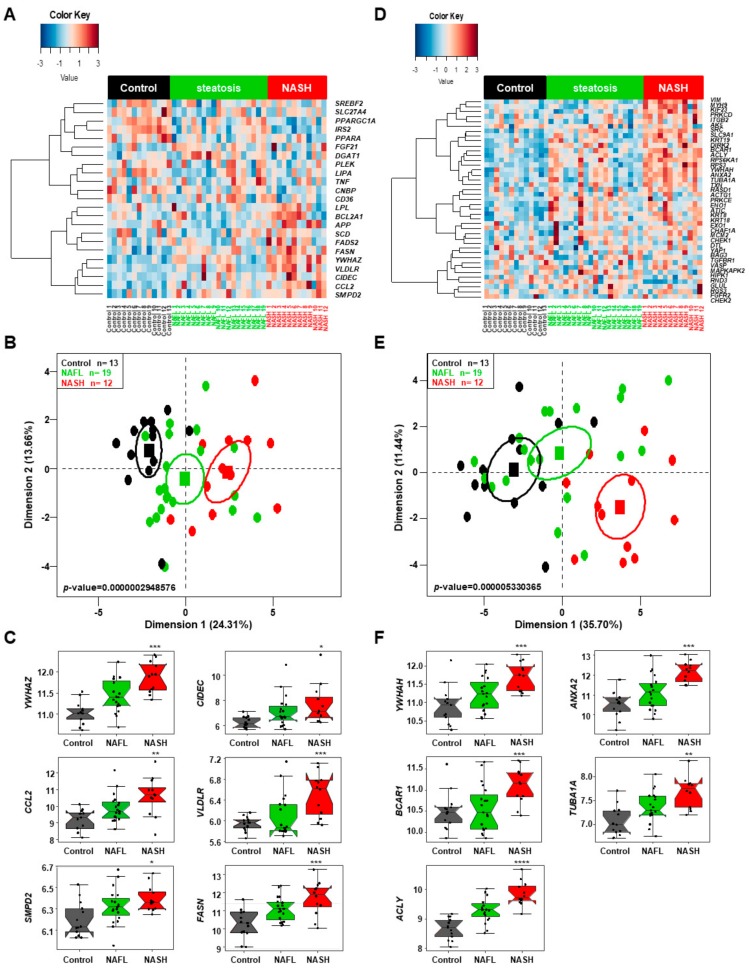
The NASH canonical pathway confirmed by independent Illumina gene expression beadchip. (**A**) Heatmap, (**B**) principal component analysis (PCA) and (**C**) boxplots of the main genes discriminating the three groups of patients of the PCA (**B**) based on the 25 genes of the canonical pathway applied to independent Illumina gene expression beadchip. (**D**) Heatmap, (**E**) principal component analysis (PCA) and (**F**) boxplots of the main genes discriminating the three groups of patients of the PCA (E) based on the 44 genes partners of the YWHAZ proteins linked to the canonical pathway applied to independent Illumina HumanWG-6 v3.0 gene expression beadchip (GSE33814) [65]. The groups of healthy control (*n* = 13), nonalcoholic fatty liver (NAFL, *n* = 19) and nonalcoholic steatohepatitis (NASH, *n* = 12) patients are significantly different: * *p* < 0.05; ** *p* < 0.01, *** *p* < 0.005, **** *p* < 0.0005 based on ANOVA-one-way analysis.

We performed an unsupervised clustering analysis and showed that the three groups of patients are perfectly separated (Figure 8A) and from 25 genes in our established gene signature, six are significantly differentially expressed between the three groups as shown by the first dimension on the PCA analysis (Figure 8B, *p*-value *=* 0.29 × 10^-6^). The six main genes are involved in the cancer and inflammatory processes (*YWHAZ*, *CCL2* and *SMPD2*) and lipid droplet formation and metabolism (*CIDEC*, *VLDLR* and *FASN*) and significantly increased in NASH patients compared to control or NAFL groups (Figure 8C).

Then we also looked at the 44 partners of YWHAZ proteins linked to the canonical pathway. The unsupervised clustering analysis showed that the three groups of patients are perfectly separated (Figure 8D) and that among the 44 genes encoding for *YWHAZ* partners, five genes significantly discriminate among the three groups of patients on the first dimension of the PCA (Figure 8E, *p*-value *=* 5.3 × 10^-6^). The five genes emphasized that the cancer pathway is associated with a significant increase in *YWHAH* and *BRCA1* expression, as well as an increase in expression of *ACLY*, a gene implicated in the first step of the lipid metabolism. The cancer pathway is also associated with two genes implicated in the cytoskeletal remodeling and network, *ANXA2* and *TUBA1A*, linked to HCC and cell migration (i.e., metastasis development) as shown previously [66,67], thus predicting the fate of the NASH (Figure 8F).

In conclusion, we were able to confirm that the pathway that includes 25 dysregulated genes is common in patients who developed NAFL and then NASH, leading to the concept of a common signature of genes connected together that might seal the fate of NAFL to progress to NASH and then insulin-resistance, inflammation, and finally cancer. We also confirm that other genes might emphasize the fate of the NASH progression to a worst outcome in some patients.

### 2.7. Genes Implicated in Progression of NASH are Part of the Progression to Hepatocellular Carcinoma

We identified 25 genes implicated in the progressions of steatosis to NASH, particularly genes implicated in lipid metabolism, inflammation processes, and five more genes implicated in cancer development.

**Figure 9:**
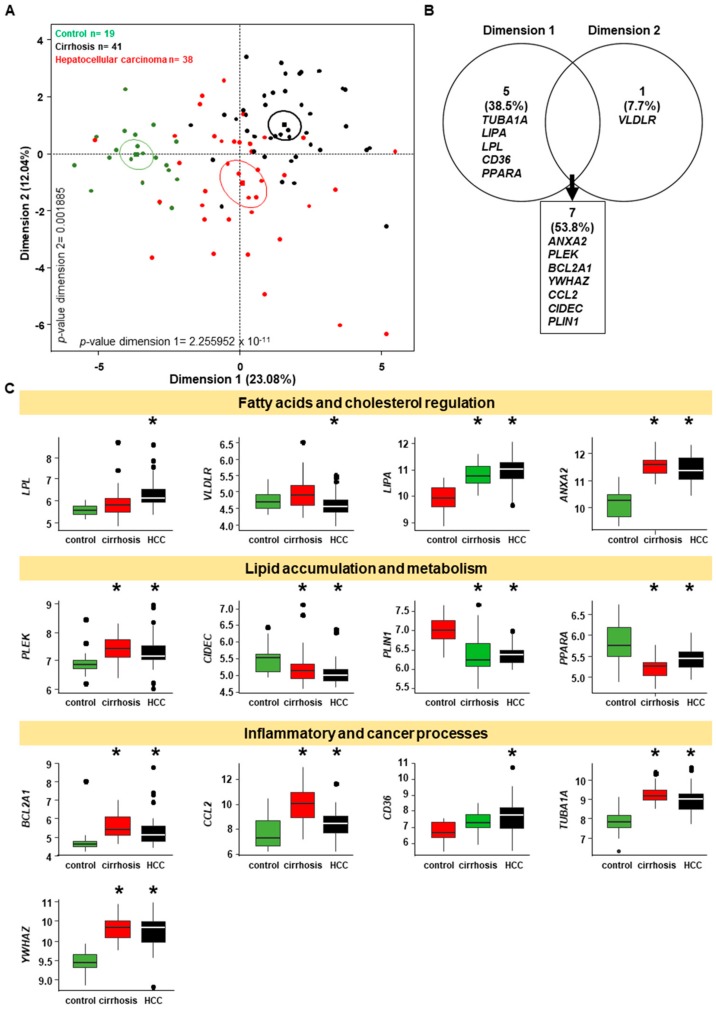
The genes involved in NASH progression are also involved in hepatocellular carcinoma. (**A**) Principal component analysis (PCA) based on the 30 genes implicated in NASH progression and run with the dataset of GSE14323 implicating control (*n* = 19), cirrhosis (*n* = 41) and HCC (*n* = 38) liver biopsies separated significantly on the first dimension and second dimension. (**B**) Venn diagram based on the genes (i.e. variables) implicated of the separation of the three groups of patients from the PCA (**A**) leading to a total of 13 genes. (**C**) Boxplots of the expression of the 13 genes in the in each group control, cirrhosis and HCC compared by ANOVA one-analysis. * *p* < 0.05.

Thus, to dissect the role of these 30 genes in the progression of NASH to HCC, we used the data provided by the GSE14323 [68] including biopsies for which gene expression was analyzed from 19 controls, 41 cirrhosis, and 38 HCC using [HG-U133A] Affymetrix Human Genome U133A Array. PCA showed that the three groups of patients separated significantly on the first dimension (*p*-value = 2.255952 × 10^-11^) and second dimension (*p*-value = 0.001885) implicating 13 genes (Figure 9A,B). These 13 genes include genes involved in regulation of fatty acid and cholesterol metabolism such as *LPL*, *VLDLR*, *LIPA*, *ANXA2*, and *PLEK*; lipid accumulation (*CIDEC*, *PLIN1*); and metabolism (*PPARA* and *BCL2A1*); and especially genes implicated in inflammatory and cancer processes such as *CCL2*, *CD36*, *TUBA1A*, and *YWHAZ* (Figure 9C). Most of the latter are significantly up-regulated in cirrhosis and hepatocellular carcinoma samples, while genes implicated in lipid accumulation such as *CIDEC* and *PLIN1* are significantly down-regulated. This may explain changes in the energy metabolism, particularly lipid metabolism, that is modified in hepatocellular carcinoma with down-regulation of triglycerides and ceramides leading to a decrease in lipid droplets due to the increase in lipid metabolism turn-over [69]. This is in concordance with what was shown less than a decade ago about hepatocellular carcinoma that can evolve from NASH [70,71].

Thus, we went further to analyze the role that 30 genes found in NASH which may play a role in the development of hepatocellular carcinoma. We investigated genomic data of a liver cancer cohort from TCGA consortium (http://www.cbioportal.org) [72]. Indeed, we used this approach because it is well known that, in cancer progression, genomic instability could affect genes through mutation but also through the copy number variation. It is also known that, in liver cancer, mutation profile acquired in the tumor tissue is not enough to explain all the classification of the cohort of patients with HCC. The average number of acquired mutations in each HCC patients could range from 50 to 60 [73].

**Figure 10:**
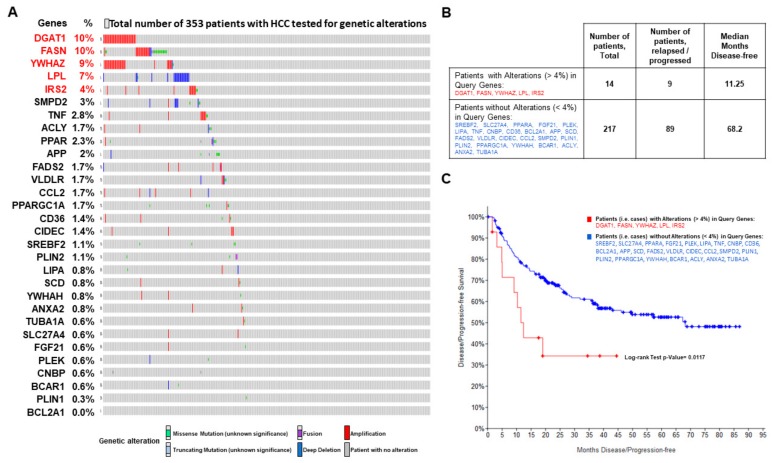
A core of 5 genes involved in NASH progression have alterations found in hepatocellular carcinoma which are associated with a very poor outcome. (**A**) Investigation of the 30 genes involved in NASH on genomic data including copy number variations, single nucleotide polymorphism, mutation, fusion and deletion of liver cancer cohort from TCGA consortium performed in 353 patients (https://www.cbioportal.org/) [72] leading to five genes present in more than 4% of patients. (**B**) Table from (A) recording the total patients with genetic alterations (≥4%) in the 5 genes of interest (*n* = 14) and the total patients (*n* = 217) without genetic alteration (<4%). Among these patients some patients (*n* = 9 and *n* = 89, respectively) have been identified with relapse or progression of the liver cancer. (**C**) Kaplan-Meier survival curve between patients (*n* = 9) with genetic alterations (≥4%) in the five genes identified in (A) and patients (*n* = 89) without (<4%) genetic alterations in the 24 other genes associated with relapsed or progressed cancer. Kaplan-Meier curves were analyzed by log-rank test.

Among the 353 patients with HCC, 231 patients have genetic alterations (e.g., mutations, fusion, deletions, amplifications, or single nucleotide polymorphisms) in 29 genes, but for one gene (*BLC2A1*) no alteration was observed in these patients based on the genomic landscape of liver HCC and mutational signatures results (Figure 10A). Interestingly, among the 29 genes, alterations in five genes -*DGAT1*, *FASN*, *YWHAZ*, *LPL*, *IRS2*- have been found in more than 4% of patients with HCC. The data analyses showed that genetic alterations included amplifications, missense mutations, and deletions. We showed that presence of mutations in these five genes in patients (*n* = 9) was associated with a higher chance of relapse or progression of the disease compared to patients (*n* = 89) with fewer alterations (less than 4% for the other 24 genes, Figure 10A,B), having a poor prognosis with a significantly shorter time without progression or recurrence (11.25 months vs. 68.2 months, respectively, Figure 10B,C, Kaplan-Meier analysis with log-rank test *p*-value = 0.0117).

In summary, we have shown that the genes previously identified as key players in the NASH progression were also implicated in liver cancer. These genes were involved in lipid metabolism and regulation, inflammation, and cancer development. Changes in expression of these genes are linked to the progression of NASH to cancer. Additionally, genetic alterations in these genes are strongly involved, especially mutations in five genes (*DGAT1*, *FASN*, *LPL*, *IRS2*, and *YWHAZ*), in lipid synthesis, insulin resistance, and cancer progression. Therefore, these genomic alterations playing gene regulatory role for driving the progression of HCC has been shown to be significant in NASH progression.

## 3. Discussion

Since we began this study, more than 2,550,897 papers have been published in PubMed but only 4916 new articles about NAFLD were released, and 0.197% of these publications included the following fields such as reviews; original articles focused on diagnosis; treatment; new marker(s) and cofactors; epidemiology; cause or relationship with other diseases, i.e., cardiovascular disease, fibrosis, muscular dysfunction, drug-induced NAFLD; and some articles focused on mechanisms (source from https://www.ncbi.nlm.nih.gov/). In general, manuscripts that focused on mechanisms, attempt to confirm or disprove previous data leading to controversial results [74,75] while adding additional data. However, to date, no comprehensive study, combining all the relevant data to find a canonical mechanism implicated in NAFL/NASH progression, has been performed. In the last few years, as bioinformatic tools were developed to perform data-mining and text-mining, laboratories in NAFLD field have started to use these approaches to develop a diagnostic tool based on a decision tree using a machine learning approach [76]. These tools have also been used to develop an algorithm that would perform data mining [77] to predict NAFLD-cancer progression, or to find a specific signature and mechanism of NASH progression using lipidomic data [19].

Thus, in this manuscript we used text-mining and data-mining based approaches and found a NAFLD/NASH canonical mechanism summarized in Figure 7.

Indeed, the text mining approach reduced the transcriptomic matrix to the most important genes highlighted by scientific literature. This mathematical approach to reduce the dimension had a major advantage as compared to classical analysis which was also to reduce the false discovery error link to the high dimension data of the transcriptome. During transcriptome analysis, usual statistical approach applied for more than 30,000 variables and for each row is to use 5 percent of false positive error of discovery, with some corrections applied like FDR or BH, usually introduced to minimize this trouble. By this conventional approach, if the biological information is small and diluted in unrelated experimental variance, the biological information could be totally ignored by the statistical hypothesis. The advantage of our work is to focus on the statistical hypothesis to a few hundred genes and so to considerably reduce the risk to the unseen biological effect, especially in the context of liver, in which the biology and the metabolism are very complex.

For some selected genes, in the case of the gene co-occurrences which were biased in the scientific literature, the second step of our workflow was to eliminate these unrelated genes because literature information was confirmed in transcriptome data from patients with NAFLD.

During this work, a protein–protein interaction network was built around significant genes. This bioinformatic step enlarged the literature approach by the fact that the initial gene analysis was introduced in neighbor molecules which were experimentally connected to the initial selected markers from the scientific literature. This original bioinformatic approach allowed identification of a canonical mechanism for the progression from NAFL to NASH and then, probably in some patients, the progression from NASH to cirrhosis/HCC.

As expected, four major axes have been identified validating our approach. The first axe concerns lipid metabolism dysregulation including increased lipid influx, increased de novo lipid synthesis, and decreased mitochondrial function that leads to fat accumulation in the form of intracellular lipid droplets. The second pathway includes inflammatory processes implicating lipids and chemo-attracting molecules which lead to recruitment of inflammatory cells such as macrophages. The third pathway, which is activated as a result of activation of the previous two, is the insulin resistance pathway. All these three pathways lead to the development of liver cancer. What is interesting in our analysis is that we were able to connect all these pathways together showing key genes and pathways that connect all these processes. However, we should consider this canonical mechanism as a nucleus and not as a dogma. For instance, recently we and others, using different approaches, have shown that *FADS1* (i.e., Δ5-desaturase) polymorphism can decrease enzyme activity leading to the accumulation of toxic fatty acids upstream in the pathway and decrease the downstream phosphatidylcholine to phosphatidylethanolamine ratio in NASH patients, leading to hepatocyte death and release of lipids that are toxic for the surrounding hepatocytes [19,20,35]. Thus, *FADS1* can be incremented in the canonical pathway summarized in Figure 7. We should consider that in all NASH patients, activation of this canonical pathway is a common feature, and some of patients can have additional traits (e.g., *FADS1* polymorphisms/decrease enzyme activity) that increase their risk for a faster progression of the disease from NAFL to NASH and/or from NASH to HCC. Thus, each additional trait, such as *PNPLA3* and/or *FADS1* polymorphisms, can be incorporated into the canonical pathway to build a more complex mechanism and then to have a larger overview of the mechanism implicated.

Also, in using this canonical pathway as a main foundation to study NAFLD progression, researchers will be able to have a new angle to understand the disease and to find new treatment(s) or approaches to treat NASH-patients. Recently, Musso G et al. published an interesting review focusing on bioactive lipid species and metabolic pathways implicated in NASH progression based on 162 publications. This resulted in description of general mechanisms, but did not identify the most critical mechanism among all of these mechanisms or how the connections between these mechanisms are made [78]. The general mechanisms described in this review are connected to the canonical pathway that we found but with more detail about which partners are important.

The study by Muss G et al. [78] and our study do not address the assessment of the probability weight of the risk of having particular polymorphisms (e.g., *FADS2* polymorphisms vs. *FADS1* polymorphisms vs. *PNPLA3* polymorphisms) or to have metabolic dysregulations of lipids from the diet, for instance. Defining an odds ratio or risk ratio for each element of the canonical pathway and the other pathways will be the next step to understand the imbalance that occurs during NAFLD progression.

This study also showed for the first time the role of proteins *YWHAZ* and *YWHAH* which belong to the 14-3-3 protein family. Both proteins YWHAZ and YWHAH are implicated in HCC progression and metastasis. Indeed, the *YWHAZ* gene is well known to be up-regulated in HCC patients but now *YWHAZ* is identified as an oncogene based on recent research on Cancer Genome Atlas [60] and implicated in mitochondrial function [79]. *YWHAH* has also been implicated in liver cancer depending on regulated c-myc expression [63], in insulin resistance [62] and mitochondrial function [80].

Finally, to make the final connection between genes implicated in NASH progression and hepatocellular carcinoma development, we investigated the genes in two genome datasets including normal, cirrhotic, and HCC patients (GSE14323) and also 353 patients with HCC for which copy number variations and SNP were identified. We found five genes associated with poor prognosis including *FASN*, *DGAT1*, *LPL*, *IRS2*, and *YWHAZ*. Four of these genes are implicated in lipid metabolism regulation and one in liver cancer. Recently, lipid metabolism reprogramming in hepatocellular carcinoma has become the focus of research [81]. Several studies have shown that knockdown or pharmacological inhibition of FASN suppressed the growth of HCC in vitro. In vivo, genetic ablation of FASN completely suppressed Akt-driven HCC development through the inhibition of Rictor/mTORC2 signaling [81,82]. Recently, another in vivo study confirmed the previous findings and showed that genetic deletion of FASN totally suppresses hepatocarcinogenesis driven by AKT and AKT/c-Met protooncogenes in mice. On the other hand they showed also that liver tumor development is completely unaffected by FASN depletion in mice co-expressing β-catenin and c-Met strongly suggesting that lipid metabolism could play a role not directly in the development of the HCC but in the prognosis of the HCC progression [83]. Indeed, it has been shown that FASN is frequently up-regulated in various cancers, and its increased expression is associated with chemoresistance, metastasis, and poor prognosis [81]. It has been shown that LPL is also up-regulated in mouse and human HCC associated with up-regulation of FASN [84]. In addition, IRS2 has been shown to be overexpressed in murine and human HCC and participate in the development of the disease with IRS1 through AKT pathway [85,86]. Thus, these five genes make the core of the canonical pathway. We also showed that not only dysregulation of their expression but also genetic alterations in these genes play an important role in the progression of the disease.

Our results are in accordance with a recent discovery showing a twist in lipid metabolism in hepatocellular carcinoma [87,88]. Indeed, in this paper they showed that many cancer cells activate lipid-synthesis pathways to support their rapid proliferation, especially hepatocellular carcinoma implicating two enzymes SCD and FADS2. They showed that some types of cancer cell are insensitive to modifications of SCD and continued to grow implicating a second enzyme FADS2. They showed that in HCC, FADS2 uses palmitate like SCD but produces sapienate (instead of palmitoleate), a monounsaturated fatty acid produced in sebaceous gland that will be incorporated into the phospholipids in the membrane of the liver cancer cell to adapt its needs to survive and to proliferate [88]. The tumor environment such as fibrosis, hypoxia, dysregulated metabolism might also influence the liver cancer cell regulation of SCD and FADS2 enzyme activities especially in the case of HCC developed from NASH stage [81]. Indeed, *SCD*, *FADS1*, and *FADS2* are dysregulated in NASH as we showed in this study and previously [19,20,81].

Finally, to go further into the understanding of the genes implicated in the canonical pathway described in this study, the next step will be first to test this canonical pathway in a different new cohort of patients with steatosis and NASH associated to different genetic backgrounds and environments in prospective studies. Afterwards, it will be to perform single-cell analysis in different liver biopsies from healthy lean and obese patients, lean and obese patients with NAFL, NASH, cirrhosis, and HCC developed from NASH. The cells that should be analyzed will be at least hepatocytes, cholangiocytes, hepatic stellate cells, endothelial cells, and Kupffer cells. Indeed, in our study we used dataset from liver biopsies reflecting the bulk liver RNA. Currently, such single cell analyses in the different groups of patients mentioned above have not been performed yet.

**Figure 11:**
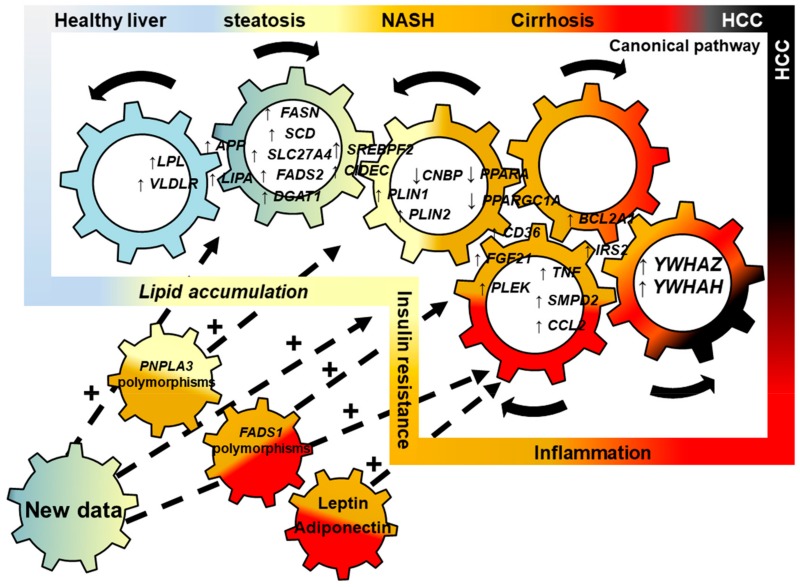
Canonical pathway explaining the NASH progression from steatosis to hepatocellular carcinoma. Inside the box the genes identified by text-mining are represented. The toothed wheels represent the connections between the genes working together to accumulate toxic lipids that will lead to insulin-resistance and then inflammation and hepatocellular carcinoma (HCC). Thus, the steatosis due to an imbalance of lipid metabolism and accumulation of toxic lipids will progress to NASH due to the inflammatory processes and insulin resistance leading to expression of genes involved in tumor progression such as YWHAZ and YWHAH. Outside the box, other genes with specific polymorphisms (e.g., FADS1: fatty acid desaturase 1) or other factors such as cytokines/chemokines (e.g., increase of leptin and/or decrease of adiponectin) will accelerate and/or exacerbate the progression of the disease leading faster to cirrhosis and then HCC. This representation is not frozen but dynamic and can change depending on the future new data.

In conclusion, using an original approach based on text-mining and data-mining we were able to identify 25 genes implicated in NASH progression and 44 genes/proteins implicated in progression of the disease from NASH to HCC (Figure 11). This analysis highlighted genes belonging to the 14-3-3 protein family *YWHAZ* and *YWHAH*. Both proteins YWHAZ and YWHAH are implicated in cancer progression, especially in liver cancer. Thus, this might explain why some patients with NASH may or may not progress to HCC.

Taken together these data led to discovery of a canonical pathway for NASH progression that connects together dysregulation of lipid metabolism, inflammatory processes, insulin resistance, and cancer progression (Figure 11). In addition, other genes with specific polymorphisms or other factors such as cytokines/chemokines could accelerate and/or exacerbate the progression of the disease leading to cirrhosis and HCC faster. This canonical pathway is not frozen but dynamic and is likely to change depending on the future new data that can be integrated with the pathway. These genes should be sought in future prospective clinical studies involving patients with NAFLD.

## 4. Material and Methods

### 4.1. Bioinformatics and Statistical Analyses

#### Text-Mining Approach

Bioinformatics of gene expression analysis was approached by dimensional reduction of gene expression matrix by text-mining approach. Workflow of this analysis is described in Figure 1. Text-mining based on MeSH, a natural language processing, allowed us to found connections between language terms and gene identifiers in scientific literature such as PubMed database. Co-occurrence quantification by this approach allowed to connect gene databases to scientific literature and to highlighted important scientific relations with small set of molecules [55]. Mathematical dimensional reduction of transcriptome matrix was focused on significant genes found connected to literature after false discovery rate (FDR) correction in order to minimized false positive discovery and set-up below 5% [89].

Lipid genes differential expressed between NASH, healthy obese, and NAFL obese liver samples were searched with Significance Analysis for Microarray (SAM) algorithm by implementing FDR threshold under 5 percent [90]. Unsupervised PCA was performed with “*FactoMineR*” R-package and group discrimination *p*-value was estimated with variable correlation to the first principal component axis [91,92].

Functional enrichment analysis was performed with the standalone software GO-Elite version 1.2 [93] on Gene Ontology-Biological Process (GO-BP) and the Comparative Toxicogenomics Database (CTD). Functional enrichment networks were built with Cytoscape software version 3.0. [94] with information collected during functional enrichment: the blue edge represents connections between genes and functions, blue circle nodes represent enriched genes, and octagon nodes represent enriched functions, scale color from yellow to purple in the function nodes is proportional to the Z-scores obtained during the enrichment. PubMed gene prioritization by connection to the NCBI website was performed with java application Gene Valorization working under Java Virtual Machine [95]. Gene prioritization relations with scientific literature were represented as a Circos plot with “*circlize*” R-package [92,96].

### 4.2. Transcriptome Dataset Analysis Narrowing Text-Mining Discovery

Transcriptome dataset of liver samples included HO subjects (*n* = 24), NAFL obese patients (*n* = 23), and samples of patients affected by NASH (*n* = 24) [61]; it was downloaded from Gene Expression Omnibus database under accession number GSE61260 (https://www.ncbi.nlm.nih.gov/geo/query/acc.cgi?acc=GSE61260). These experiments were performed with Affymetrix technology and microarray version Human Gene 1.1 ST array. Normalized matrix by robust multi-array average (RMA) algorithm [97] was merged on identifier column with corresponding annotation platform GPL11532 (https://www.ncbi.nlm.nih.gov/geo/query/acc.cgi?acc=GPL11532) to create an *in silico* experimental matrix. This matrix comprises 71 patients divided in three groups for which each liver sample hybridized array comprised more than 750,000 unique 25-mer oligonucleotide probes that interrogate more than 28,000 genes.

Then, receiver operating characteristic (ROC) curves with area under the curve (AUC) performed on altered lipid genes were done with “*pROC*” and “*Epi*” R-packages [98,99]. Boxplots, Kruskal-Wallis test, and Student *t*-test were performed in R software environment version 3.4.3 [92].

The patient sample characteristics from GSE61260 dataset can be found in the paper published by Horvath et al. on the Supplementary Information of the paper [61].

Briefly, patients were only Caucasians from Germany. RNA was extracted from human liver samples and analyzed as described above.

Patients were divided in different groups such as NAFL (*n* = 23), NASH (*n* = 24), HO subjects (*n* = 24) based on the results of the histology analysis performed by a pathologist. Liver samples were obtained percutaneously for patients undergoing liver biopsy for suspected NAFLD or intraoperatively for assessment of liver histology.

The three groups of patients were clustered based on the total NAFLD Activity Score (NAS) [21,100]. Briefly, the total NAS represents the sum of scores for steatosis, lobular inflammation, and hepatocyte ballooning, and ranges from 0 to 8 [21,100]. After diagnosis, NASH or fatty liver not diagnostic of NASH, the total NAS is used to grade activity. NAS scores of 0–2 typically occur in cases largely considered not diagnostic of NASH, whereas scores of 5–8 usually occurs in cases that are considered diagnostic of NASH. Steatosis: ordinal variable that relates to the amount of surface area involved by steatosis as evaluated on medium power examination. Minimal steatosis (<5%) receives a score of 0. 5–33% (score of 1), 33–66% (score 2), and >66% (score 3). Liver inflammation: ordinal variable: 0 corresponds to no foci, 1 (<2 foci/200×), 2 (2–4 foci/200×), 3 (>4 foci/200×). Fibrosis: ordinal variable that takes on (half) integer values between 0 and 4: 0 (none), 2 (perisinusoidal and portal/periportal), 3 (bridging fibrosis), 4 (cirrhosis). The fibrosis stage is evaluated separately from the total NAFLD score.

Hepatocyte ballooning was measured in each biopsy as follow: ballooning: 0 (none), 1 (few balloon cells). Here “few” means rare but definite ballooned hepatocytes as well as cases that are diagnostically borderline, 2 (many cells/prominent ballooning).

Patients were also checked for free-hepatitis B or C virus infections.

All patients provided written, informed consent. The study protocol was approved by the institutional review board (“Ethics commission of the Medical Faculty, University of Kiel”, project identification: D425/07, A111/99) before the commencement of the study, as published in the original paper [61].

### 4.3. Confirmation of NASH Canonical Pathway by Using an Independent Validation Cohort and a Different Transcriptome Technology Analysis

An independent transcriptome series was process in order to validate the inflammatory/lipid gene expression profile of the NASH canonical pathway using a published GSE33814 [65] including 44 human liver tissue surgical samples (normal *n* = 13; steatosis *n* = 19; steatohepatitis *n* = 12) which was process with Illumina HumanWG-6 v3.0 expression beadchip technology. Normalized dataset was annotated with corresponding Gene Expression Omnibus platform GPL6884. Annotated matrix was restricted to inflammatory lipidic signature by SQL querying and process to perform supervised expression heatmap with “*made4*” R-package/Bioconductor repository [101] and unsupervised PCA with “*FactoMineR*” R-package [91]. ANOVA-One way with Tukey post Hoc test was done on highlighted biomarkers in R software environment version 3.4.3 [92]. The study was approved by the ethical review committee at the University of Graz (EK number: 20-119 ex 08/09), as published in the original paper [65].

### 4.4. Genes Implicated in NASH Progression Involved in Progression to Hepatocellular Carcinoma: Liver Cancer Genomic Data

To assess the play of the genes implicated in NASH progression, we investigate the gene profile of GSE14323 [68]. Liver tissue samples were obtained from patients waiting for liver transplantation at one of the GR2HCC Centers. Additionally, normal liver and tumor samples were also obtained from the Liver Tissue Cell Distribution System. For each sample, RNA was extracted and hybridized to an Affymetrix GeneChip ([HG-U133A] Affymetrix Human Genome U133A Array) including 19 controls, 41 HCV-cirrhosis and 38 HCV-advanced hepatocellular carcinoma patients.

In the second way, we investigated genomic data (copy number variations: CNV and single nucleotide polymorphism: SNP) of liver cancer cohort from The Cancer Genome Atlas (TCGA) consortium [72] through cbioportal web application (https://www.cbioportal.org/) [102,103]. This dataset contained Tumor Samples with sequencing and CNA data (353 patients/samples). Oncoprint of the CNV and SNP comprised in this dataset for the 30 lipid-related genes were performed. The number of patients who relapsed or progressed based on their cancer genetic profile (i.e. free survival) was assessed over the time using Kaplan-Meier analysis with “*survival*” R-package and analyzed using stratified log-rank survival test. The research protocol was approved by the respective institutional review boards, and informed consent was obtained in all cases, as published in the original paper [68].

## Supplementary Materials

Supplementary materials can be found at https://www.mdpi.com/1422-0067/20/22/5594/s1. Supplementary Datasets Table S1: Text-mining list of genes associated in PubMed literature with lipid related keywords. Supplementary Datasets Table S2: Expression fold change of lipid related genes found differentially expressed between NASH and healthy obese liver samples. Supplementary Datasets Table S3: Liver as principal filter for prioritization of lipid related genes found differentially expressed in NASH. Supplementary Datasets Table S4: Gene prioritization secondary filters (immunological, inflammation, liver pathogenesis progression) table found with lipid related genes differentially expressed in NASH. Supplementary Datasets Table S5: Identification of protein partners of YWHAZ gene using InnateDB database. In red, “*YWHAZ* gene encodes for a 14-3-3 protein family and it is well known to be up-regulated in HCC.”
