# Supplementary material for: Lipid Related Genes Altered in NASH Connect Inflammation in Liver Pathogenesis Progression to HCC: A Canonical Pathway
Source: Int J Mol Sci. 2019 Nov 8;20(22):5594. doi: 10.3390/ijms20225594 (PMC6888337; doi:10.3390/ijms20225594)
Supplement: Supplementary file 1 [file ijms-20-05594-s001.pdf]

Lipid related genes altered in NASH connect inflammation in liver pathogenesis progression to HCC: a canonical pathway

Christophe Desterke[1], Franck Chiappini[2*]

[1] Inserm, U935, Villejuif, F-94800, France
[2] Cell Growth and Tissue Repair (CRRET) Laboratory, Université Paris-Est Créteil (UPEC), EA 4397 / ERL CNRS 9215, F-94010, Créteil, France.

Corresponding author: *Franck Chiappini. Laboratoire du CRRET (Croissance, Réparation et Régénération Tissulaires), Université Paris-Est Créteil, 61 avenue du Général de Gaulle F-94010, Créteil Cedex, Val de Marne, France. Email address: fchiappini@yahoo.fr; Tel: +33(0)145177080; Fax: +33(0)145171816

Supplementary Information

Supplementary Datasets Table S1: Text-mining list of genes associated in PubMed literature with lipid related keywords.

Supplementary Datasets Table S2: Expression fold change of lipid related genes found differentially expressed between NASH and healthy obese liver samples.

Supplementary Datasets Table S3: Liver as principal filter for prioritization of lipid related genes found differentially expressed in NASH.

Supplementary Datasets Table S4: Gene prioritization secondary filters (immunological, inflammation, liver pathogenesis progression) table found with lipid related genes differentially expressed in NASH.

Supplementary Datasets Table S5: Identification of protein partners of YWHAZ gene using InnateDB database.

Supplementary Datasets Table S1: Text-mining list of genes associated in PubMed literature with lipid related keywords.

Ranking of "lipidic" textmining	Gene symbol	Co-occurence (raw p-values)	FDR (q-values)
1	*SREBF1*	0.0002619114	0.01358466
2	*PPARA*	0.0003235560	0.01358466
3	*ABCA1*	0.0003681963	0.01358466
4	*LPL*	0.0003975563	0.01358466
5	*FASN*	0.0004496562	0.01358466
6	*APOA1*	0.0004580742	0.01358466
7	*SCARB1*	0.0005231800	0.01358466
8	*SCD*	0.0005678461	0.01358466
9	*APOB*	0.0006196621	0.01358466
10	*HMGCR*	0.0006470239	0.01358466
11	*APOE*	0.0006886471	0.01358466
12	*SCARB2*	0.0006988881	0.01358466
13	*CD36*	0.0007133737	0.01358466
14	*PPARG*	0.0007758856	0.01358466
15	*PLB1*	0.0009038230	0.01358466
16	*LDLR*	0.0009385414	0.01358466
17	*SMPD2*	0.0009595884	0.01358466
18	*PLA2G1B*	0.0009698991	0.01358466
19	*YWHAZ*	0.0010493838	0.01358466
20	*ADIPOQ*	0.0010539931	0.01358466
21	*SMPD1*	0.0010821174	0.01358466
22	*LCAT*	0.0011049149	0.01358466
23	*MTTP*	0.0011272159	0.01358466
24	*SREBF2*	0.0011384588	0.01358466
25	*DGAT1*	0.0011396530	0.01358466
26	*PLIN1*	0.0012719951	0.01430711
27	*HRASLS*	0.0013887410	0.01430711
28	*CNBP*	0.0014569059	0.01430711
29	*HDL3*	0.0015138986	0.01430711
30	*CPT1A*	0.0015385325	0.01430711
31	*INS*	0.0015852406	0.01430711
32	*MBTPS1*	0.0016245889	0.01430711
33	*PLIN2*	0.0016306575	0.01430711
34	*LIPE*	0.0016323545	0.01430711
35	*ABCG1*	0.0017635069	0.01495844
36	*JPH3*	0.0018070596	0.01495844
37	*NPC1*	0.0021632303	0.01722259
38	*DGAT2*	0.0021961697	0.01722259
39	*NPCA1*	0.0022545698	0.01722723
40	*SCP2*	0.0025027804	0.01831917
41	*CHPT1*	0.0025474601	0.01831917
42	*PNPLA2*	0.0027216898	0.01831917

43	*CPT1B*	0.0028029737	0.01831917
44	*LEP*	0.0028422318	0.01831917
45	*SGMS2*	0.0028485242	0.01831917
46	*ASAH1*	0.0028774565	0.01831917
47	*FGF21*	0.0028892645	0.01831917
48	*CEL*	0.0029698786	0.01835349
49	*SLC2A4*	0.0030178560	0.01835349
50	*SYCP2*	0.0032022351	0.01908532
51	*FADS2*	0.0032797107	0.01916380
52	*FABP1*	0.0034752870	0.01986396
53	*VLDLR*	0.0035328524	0.01986396
54	*LIPA*	0.0036666346	0.02008373
55	*NPC2*	0.0037067289	0.02008373
56	*PLA2G2A*	0.0039595445	0.02075329
57	*SLC27A4*	0.0039695893	0.02075329
58	*ACACA*	0.0041355263	0.02124805
59	*SPTLC2*	0.0045443596	0.02295287
60	*GOT2*	0.0046960671	0.02300699
61	*PLA2G6*	0.0047094851	0.02300699
62	*INSR*	0.0048657774	0.02338712
63	*CIDEC*	0.0052143154	0.02466454
64	*DECR1*	0.0054331306	0.02502701
65	*NPC1L1*	0.0054589118	0.02502701
66	*CAV1*	0.0061107334	0.02754835
67	*GCG*	0.0061937563	0.02754835
68	*IL6*	0.0067375943	0.02952652
69	*APOM*	0.0068660154	0.02965323
70	*PLEK*	0.0072534824	0.03037180
71	*BCL2A1*	0.0072624198	0.03037180
72	*TNF*	0.0073381539	0.03037180
73	*NR1H4*	0.0078026279	0.03185182
74	*ALB*	0.0095523640	0.03846763
75	*APP*	0.0098978354	0.03932740
76	*PCSK9*	0.0104411137	0.04094016
77	*GBA*	0.0108495909	0.04195473
78	*CAT*	0.0109814393	0.04195473
79	*KAT5*	0.0113702228	0.04239619
80	*PPARGC1A*	0.0114432670	0.04239619
81	*FDFT1*	0.0115237974	0.04239619
82	*CCL2*	0.0117295343	0.04262684
83	*MIR7-3HG*	0.0119889722	0.04304474
84	*ABCA4*	0.0122812342	0.04356914
85	*PTK2B*	0.0125874955	0.04413028
86	*SOAT1*	0.0133278609	0.04618259
87	*IRS2*	0.0144702554	0.04956478

Connections of gene symbols co-occurrence with lipid related keywords (**Fig. 2A**) with PubMed database, columns result ranking of text mining with gene symbol and respective raw p-values and corrected q-values by false discovery rate (FDR)**.**

Supplementary Datasets Table S2: Expression fold change of lipid related genes found differentially expressed between NASH and healthy obese liver samples.

Textmining ranking	gene ID	lipidic related gene		Textmining FDR q-values	SAM Fold Change
4	4023	*LPL*	lipoprotein lipase	0.01358466	1.9289734
82	6347	*CCL2*	C-C motif chemokine ligand 2	0.04262684	1.6215025
51	9415	*FADS2*	fatty acid desaturase 2	0.01916380	1.575647
26	5346	*PLIN1*	perilipin 1	0.01430711	1.466759
47	26291	*FGF21*	fibroblast growth factor 21	0.01831917	1.4664658
5	2194	*FASN*	fatty acid synthase	0.01358466	1.3429465
71	597	*BCL2A1*	BCL2 related protein A1	0.03037180	1.3212321
8	6319	*SCD*	stearoyl-CoA desaturase	0.01358466	1.2663149
70	5341	*PLEK*	pleckstrin	0.03037180	1.2354975
13	948	*CD36*	CD36 molecule	0.01358466	1.2010236
33	123	*PLIN2*	perilipin 2	0.01430711	1.1876715
54	3988	*LIPA*	lipase A, lysosomal acid type	0.02008373	1.1638035
17	6610	*SMPD2*	sphingomyelin phosphodiesterase 2	0.01358466	1.1558349
53	7436	*VLDLR*	very low density lipoprotein receptor	0.01986396	1.1436678
63	63924	*CIDEC*	cell death inducing DFFA like effector c	0.02466454	1.141971
25	8694	*DGAT1*	diacylglycerol O-acyltransferase 1	0.01358466	1.1371824
24	6721	*SREBF2*	sterol regulatory element binding transcription factor 2	0.01358466	1.1273249
19	7534	*YWHAZ*	tyrosine 3-monooxygenase/tryptophan 5-monooxygenase activation protein zeta	0.01358466	1.1203413
75	351	*APP*	amyloid beta precursor protein	0.03932740	1.0864418
57	10999	*SLC27A4*	solute carrier family 27 member 4	0.02075329	1.0844451
72	7124	*TNF*	tumor necrosis factor	0.03037180	1.0774974
28	7555	*CNBP*	CCHC-type zinc finger nucleic acid binding protein	0.01430711	0.88592005
2	5465	*PPARA*	peroxisome proliferator activated receptor alpha	0.01358466	0.8120792
87	8660	*IRS2*	insulin receptor substrate 2	0.04956478	0.78771275
80	10891	*PPARGC1A*	PPARG coactivator 1 alpha	0.04239619	0.7431901

Columns present differentially expressed genes and their text-mining parameters (rank, q-values) but also fold changes NASH versus Healthy obese for genes found significant with Significance Analysis for Microarray (SAM) algorithm under a False Discovery Rate (FDR) of 5%.

Supplementary Datasets Table S3: Liver as principal filter for prioritization of lipid related genes found differentially expressed in NASH.

Gene symbol	Gene ID	Gene description	Gene priorization_Principal filter "Liver"
LIPA	3988	lipase A, lysosomal acid type	100000
CD36	948	CD36 molecule	28440
TNF	7124	tumor necrosis factor	17287
PPARA	5465	peroxisome proliferator activated receptor alpha	5277
FASN	2194	fatty acid synthase	3439
CCL2	6347	C-C motif chemokine ligand 2	1348
PLIN1	5346	perilipin 1	1252
APP	351	amyloid beta precursor protein	1226
LPL	4023	lipoprotein lipase	969
SCD	6319	stearoyl-CoA desaturase	802
PPARGC1A	10891	PPARG coactivator 1 alpha	616
SREBF2	6721	sterol regulatory element binding transcription factor 2	492
FGF21	26291	fibroblast growth factor 21	439
IRS2	8660	insulin receptor substrate 2	383
SLC27A4	10999	solute carrier family 27 member 4	268
DGAT1	8694	diacylglycerol O-acyltransferase 1	264
BCL2A1	597	BCL2 related protein A1	223
CNBP	7555	CCHC-type zinc finger nucleic acid binding protein	120
FADS2	9415	fatty acid desaturase 2	112
PLEK	5341	pleckstrin	93
PLIN2	123	perilipin 2	92
CIDEC	63924	cell death inducing DFFA like effector c	57
VLDLR	7436	very low density lipoprotein receptor	56
YWHAZ	7534	tyrosine 3-monooxygenase/tryptophan 5-monooxygenase activation protein zeta	30
SMPD2	6610	sphingomyelin phosphodiesterase 2	13

This table presents absolute number of PubMed articles found connected between principal filter term “liver” and Gene Symbols in NCBI website.

Supplementary Datasets Table S4: Gene prioritization secondary filters (immunological, inflammation, liver pathogenesis progression) table found with lipid related genes differentially expressed in NASH.

Gene symbol	Gene ID	Gene description	Stroma	Hepatocellular carcinoma	Liver cancer	Immuno-modulation	Inflammation	T-lymphocyte	B-lymphocyte	Hepatic macrophage	Cirrhosis
LIPA	3988	lipase A, lysosomal acid type	1204	33185	97822	6908	17669	12964	3490	4744	25442
CD36	948	CD36 molecule	41	623	2460	40	2811	123	24	506	2488
TNF	7124	tumor necrosis factor	33	1419	3338	575	5267	1447	149	1092	2149
PPARA	5465	peroxisome proliferator activated receptor alpha	1	248	658	10	745	20	8	91	399
FASN	2194	fatty acid synthase	6	367	807	90	332	379	29	67	281
CCL2	6347	C-C motif chemokine ligand 2	7	65	176	23	701	108	9	202	250
PLIN1	5346	perilipin 1	6	145	408	27	75	21	7	8	215
APP	351	amyloid beta precursor protein	0	58	219	10	127	8	2	8	159
LPL	4023	lipoprotein lipase	1	20	60	4	21	3	0	11	11
SCD	6319	stearoyl-CoA desaturase	0	33	86	2	81	4	2	8	40
PPARGC1A	10891	PPARG coactivator 1 alpha	0	15	58	0	62	0	0	5	20
SREBF2	6721	sterol regulatory element binding transcription factor 2	0	18	36	1	32	0	0	12	13
FGF21	26291	fibroblast growth factor 21	0	16	35	1	51	1	1	2	18
IRS2	8660	insulin receptor substrate 2	0	20	39	1	27	1	0	4	6
SLC27A4	10999	solute carrier family 27 member 4	1	10	50	4	13	5	1	1	26
DGAT1	8694	diacylglycerol O-acyltransferase 1	0	7	14	2	11	1	0	1	10
BCL2A1	597	BCL2 related protein A1	0	10	24	0	17	1	0	0	12
CNBP	7555	CCHC-type zinc finger nucleic acid binding protein	0	7	24	1	4	0	0	0	15
FADS2	9415	fatty acid desaturase 2	0	6	11	0	9	1	0	2	2
PLEK	5341	pleckstrin	0	8	13	0	10	2	0	3	15
PLIN2	123	perilipin 2	0	4	8	0	12	0	0	3	6
CIDEC	63924	cell death inducing DFFA like effector c	0	2	9	0	7	0	0	3	5
VLDLR	7436	very low density lipoprotein receptor	0	2	6	0	5	0	0	2	2
YWHAZ	7534	tyrosine 3-monooxygenase/tryptophan 5-monooxygenase activation protein zeta	0	6	11	0	1	1	0	0	2
SMPD2	6610	sphingomyelin phosphodiesterase 2	0	0	2	0	5	0	0	0	0

This table presents absolute number of PubMed articles found connected between secondary filter terms (related to immunity, inflammation, liver pathogenesis progression, see network on **Fig. 2A**) and Gene Symbols in NCBI website.

Supplementary Datasets Table S5: Identification of protein partners of *YWHAZ* gene using InnateDB database.

Gene Symbol	Human Symbol	Gene ID	Fold change NASH/NAFLD (FDR<5%)
RASD1	RASD1 (ras related dexamethasone induced 1)	51655	2.133833
DTL	DTL (denticleless E3 ubiquitin protein ligase homolog)	51514	1.5344161
RND3	RND3 (Rho family GTPase 3)	390	1.4639555
CHEK1	CHEK1 (checkpoint kinase 1)	1111	1.439589
MCM2	MCM2 (minichromosome maintenance complex component 2)	4171	1.4149319
ANXA2	ANXA2 (annexin A2)	302	1.3347869
KRT8	KRT8 (keratin 8)	3856	1.2938585
EXO1	EXO1 (exonuclease 1)	9156	1.2881799
KRT18	KRT18 (keratin 18)	3875	1.2777967
KRT19	KRT19 (keratin 19)	3880	1.2739475
TUBA1A	TUBA1A (tubulin alpha 1a)	7846	1.2676191
BAG3	BAG3 (BCL2 associated athanogene 3)	9531	1.2251607
TUBA1B	TUBA1B (tubulin alpha 1b)	10376	1.2251524
ACTG1	ACTG1 (actin gamma 1)	71	1.2248439
TXN	TXN (thioredoxin)	7295	1.2159586
PRKCE	PRKCE (protein kinase C epsilon)	5581	1.2022171
TUBA1C	TUBA1C (tubulin alpha 1c)	84790	1.1984481
ITGB2	ITGB2 (integrin subunit beta 2)	3689	1.1977487
VIM	VIM (vimentin)	7431	1.1840867
YWHAH	YWHAH (tyrosine 3-monooxygenase/tryptophan 5-monooxygenase activation protein eta)	7533	1.1791178
TUBB4B	TUBB4B (tubulin beta 4B class IVb)	10383	1.1693951
FGFR2	FGFR2 (fibroblast growth factor receptor 2)	2263	1.1689284
CHAF1A	CHAF1A (chromatin assembly factor 1 subunit A)	10036	1.1592104
KIF23	KIF23 (kinesin family member 23)	9493	1.1525497
RPS6KA1	RPS6KA1 (ribosomal protein S6 kinase A1)	6195	1.1468476
BCAR1	BCAR1 (BCAR1. Cas family scaffolding protein)	9564	1.1372923
YAP1	YAP1 (Yes associated protein 1)	10413	1.1360711
GLUL	GLUL (glutamate-ammonia ligase)	2752	1.1330206
ENO1	ENO1 (enolase 1)	2023	1.1319178
RGS3	RGS3 (regulator of G protein signaling 3)	5998	1.1310294
MAPKAPK2	MAPKAPK2 (mitogen-activated protein kinase-activated protein kinase 2)	9261	1.1309035
VASP	VASP (vasodilator-stimulated phosphoprotein)	7408	1.1298047
DYRK2	DYRK2 (dual specificity tyrosine phosphorylation regulated kinase 2)	8445	1.1291803
ATIC	ATIC (5-aminoimidazole-4-carboxamide ribonucleotide formyltransferase/IMP cyclohydrolase)	471	1.1272038
CHEK2	CHEK2 (checkpoint kinase 2)	11200	1.1254784
SLC9A1	SLC9A1 (solute carrier family 9 member A1)	6548	1.1216749
TGFBR1	TGFBR1 (transforming growth factor beta receptor 1)	7046	1.1199669
RPS3	RPS3 (ribosomal protein S3)	6188	1.1193616
ACLY	ACLY (ATP citrate lyase)	47	1.1182404
PRKCD	PRKCD (protein kinase C delta)	5580	1.1141334
SRC	SRC (SRC proto-oncogene. non-receptor tyrosine kinase)	6714	1.1139824
MYH9	MYH9 (myosin heavy chain 9)	4627	1.1134421
HIPK1	HIPK1 (homeodomain interacting protein kinase 1)	204851	1.0979714
AK5	AK5 (adenylate kinase 5)	26289	1.0967954

Significance analysis of microarray (SAM) of the 399 protein partners performed on GSE61260[60] identified 44 genes which discriminated perfectly both NAFL patients and NASH patients. False Discovery Rate (FDR) of 5%.
